# Calcium Carbonate Coating of 3D-Printed PLA Scaffolds Intended for Biomedical Applications

**DOI:** 10.3390/polym15112506

**Published:** 2023-05-29

**Authors:** Ricardo Donate, Rubén Paz, Álvaro Quintana, Pablo Bordón, Mario Monzón

**Affiliations:** Departamento de Ingeniería Mecánica, Grupo de Investigación en Fabricación Integrada y Avanzada, Universidad de Las Palmas de Gran Canaria, Campus Universitario de Tafira s/n, 35017 Las Palmas, Spain

**Keywords:** biomaterials, additive manufacturing, surface coating, ceramic additives, bone tissue engineering

## Abstract

The incorporation of ceramic additives is the most commonly used strategy to improve the biofunctionality of polymer-based scaffolds intended for bone regeneration. By embedding ceramic particles as a coating, the functionality improvement in the polymeric scaffolds can be concentrated on the cell–surface interface, thus creating a more favourable environment for the adhesion and proliferation of osteoblastic cells. In this work, a pressure-assisted and heat-induced method to coat polylactic acid (PLA) scaffolds with calcium carbonate (CaCO_3_) particles is presented for the first time. The coated scaffolds were evaluated by optical microscopy observations, a scanning electron microscopy analysis, water contact angle measurements, compression testing, and an enzymatic degradation study. The ceramic particles were evenly distributed, covered more than 60% of the surface, and represented around 7% of the coated scaffold weight. A strong bonding interface was achieved, and the thin layer of CaCO_3_ (~20 µm) provided a significant increase in the mechanical properties (with a compression modulus improvement up to 14%) while also enhancing the surface roughness and hydrophilicity. The results of the degradation study confirmed that the coated scaffolds were able to maintain the pH of the media during the test (~7.6 ± 0.1), in contrast to the pure PLA scaffolds, for which a value of 5.07 ± 0.1 was obtained. The ceramic-coated scaffolds developed showed potential for further evaluations in bone tissue engineering applications.

## 1. Introduction

Bone tissues affected by degenerative, surgical, or traumatic processes can be regenerated by applying bone tissue engineering (BTE) techniques, based on a combination of cells, biocompatible constructs, and bioactive molecules. One of the most promising BTE approaches is the implantation of porous biodegradable scaffolds that mimic the composition of the extracellular matrix of bone tissue, resemble its structure, and possess sufficient mechanical properties to support the tissue during its growth [1]. Taking into account the requirements for bone scaffolds, additive manufacturing (AM) techniques constitute a powerful tool for BTE applications, since they allow us to obtain tissue-engineered 3D structures with a specific design based on the needs of the patient and surgeons for their implantation. AM techniques offer great control over the pore size, pore shape, and porosity of the scaffolds, which can be tuned to mimic the function of native bone tissue [2].

Some of the most commonly used AM technologies in the biomedical field (classified by categories according to ISO/ASTM 52900:2021) include the following [3]: vat photopolymerization with curing by ultraviolet laser beam exposure (VPP-UVL, commonly known as stereolithography or SLA); vat photopolymerization with curing by exposure to ultraviolet light selectively shining through a mask (VPP-UVM, commonly known as digital light processing or DLP); and material extrusion (MEX) of low-melting-point thermoplastic materials (commonly known as fused deposition modeling or FDM). Another MEX process that has been recently gaining attention for biomedical applications is 3D bioprinting, in which soft hydrogels are used as base materials for the developed 3D structures [4]. In the specific case of BTE applications, the MEX of polymeric-based materials is one of the preferred techniques for bone scaffold manufacturing and one of the most widely used AM techniques for scaffold fabrication in general, as it allows us to obtain 3D- or 4D-printed structures with multi-material compositions and complex designs [5]. Due their advantageous properties in terms of biocompatibility, biodegradability, low toxicity, and adequate mechanical properties, coupled with the great design flexibility that they offer, synthetic polymers have attracted significant attention as base materials for bone scaffold manufacturing [6].

Among synthetic polymers, polylactic acid (PLA) has been extensively used as a base material for scaffold manufacturing in the BTE field, mainly due to its biocompatibility, biodegradability, suitable mechanical properties, tuneable degradation rate, and good processability by AM techniques, especially by material extrusion (MEX) processes [7,8]. The biomedical application of PLA scaffolds, however, is hindered by the low hydrophilicity of their surface, the lack of reactive side chain groups, and the release of acidic degradation byproducts, which can lead to a strong inflammatory response and affect tissue regeneration [9]. Inflammatory reactions at the implantation site have also been found when testing PLA samples with a high molecular weight, due to the relatively long time required for their complete degradation in vivo [10,11]. Therefore, low-molecular-weight PLA samples with a higher degradation rate are preferred in BTE to match the rate of new tissue ingrowth [12]. Other methods to accelerate PLA degradation include the following [11]: surface modification, blending, copolymerization, and compounding. In relation to the last strategy mentioned, Zhao et al. [13], developed PLA-based composite scaffolds containing magnesium (Mg) particles to increase the degradation rate of the matrix. The Mg particles were also able to neutralize the acidic byproducts of PLA, induce apatite formation, and enhance the adhesion and proliferation of osteoblast-like cells. These effects were possible as the additive simultaneously affected the surrounding media, the degradation byproducts, and the crystallinity of the base material.

On the other hand, methods proposed in the literature to enhance the biofunctionality of PLA scaffolds include the following: the incorporation of additives to the PLA matrix [14,15,16], the application of surface treatments to the 3D structure [17,18,19], and the use of bioactive coatings [20,21,22]. Among these strategies, the most commonly applied is the use of additives, and bioceramics materials have a greater potential to improve the properties of the polymer-based structure, mainly because of their bone-like composition, osteoinductivity, and biodegradability. Ceramic materials, such as hydroxyapatite (HAp), beta-tricalcium phosphate (β-TCP), ceramic bioglasses, or other calcium phosphate (CaP) compounds, have also shown a capacity to act as buffering agents during the PLA degradation process [23], enhance the mechanical properties of the matrix [24], and promote cell proliferation [25].

Examples of composite PLA-based scaffolds containing ceramic particles include those manufactured by AM (using composite materials developed by extrusion compounding) [16,26,27], solvent-based methods [28,29], thermally induced phase separation techniques [30,31], or freeze-drying processes [32,33]. In these cases, the composite material is first prepared by mixing the additive with the polymer and then processed to obtain the scaffold. Therefore, the modifications introduced by the ceramic additives affect the bulk properties, altering the structural, mechanical, and degradation characteristics of the base material. As the degradation of the PLA matrix progresses, the exposure of the ceramic particles embedded inside the matrix helps sustain a certain level of bioactivity. However, bulk modifications have a reduced effect on the functionality of the material surface, as the additives are not concentrated at the cell–surface interface but are distributed throughout the structure [17].

On the other hand, composite scaffold manufacturing strategies involving the incorporation of ceramic particles as a coating to the polymeric 3D structure can be found in the literature. Using this approach, a greater enhancement of functionality is generated on the surface of the scaffold, leading to a more favourable environment for cells during their adhesion and the early phases of proliferation [17,34]. Once surface degradation begins, the coating effect is rapidly lost, so by this time, the cells should be mature enough to support their own growth. Additionally, in contrast to bulk modifications, surface modifications can be adjusted to not affect the mechanical properties or the degradation characteristics of the original scaffold, while at the same time improving the biological properties of the surface.

According to the literature, the method to apply a ceramic coating to PLA scaffolds involves the previous application of an alkali surface treatment [21,35,36,37,38]. Chen et al. [38], for example, developed 3D-printed PLA scaffolds treated with an ammonia solution and then coated with nano-HAp particles. The alkali treatment was intended to introduce hydroxyl and carboxyl functional groups into the polymeric surface, thus favouring the binding of the ceramic nanoparticles to the PLA matrix. A 6 h alkali treatment proved to be effective in improving the surface hydrophilicity, HAp coating, and cell response. While alkali hydrolysis is a simple and cost-effective method to induce nucleation sites on PLA surfaces [19], it can also have a negative effect on the surface morphology, the mechanical properties, or the degradation characteristics of the polymeric matrix. However, the previous activation of the surface is not strictly necessary to coat PLA scaffolds with ceramic materials, as demonstrated for example by Kim et al. [39], who proposed a solution-based technique to coat PLA scaffolds with a CaP thin layer. The AM-manufactured scaffolds were soaked into a supersaturated CaP solution and stirred for 50 min. The temperature was initially set at 4 °C to allow for crystal nucleation and then increased to 37 °C to induce crystal growth. The adhesion, proliferation, and osteoblastic differentiation of rat bone marrow mesenchymal stem cells were greatly improved by the presence of the produced CaP layer. Despite the positive biological results obtained, a uniform in-depth coating and an adequate interface bonding strength are still lacking in this type of biomimetically coated scaffold [40].

In contrast, the pressure-assisted and heat-induced method proposed by Tajik et al. [34] led to uniformly coated scaffolds with a superior interface bonding strength compared to that of scaffolds coated following a biomimetic approach. The method developed consisted of five steps: ceramic slurry infiltration into the scaffold by means of an electromechanical universal tester; the subsequent dehydration of the slurry when a certain pressure is reached; the compaction of ceramic particles around the scaffold’s strands; a heat treatment step to embed the particles into the polymeric matrix; and finally, a washing step to eliminate the particles not attached to the surface. Moreover, a β-TCP coating applied to the polymeric 3D-printed scaffolds, in this case made of polycaprolactone (PCL), allowed them to obtain increased surface roughness, hydrophilicity, and cell attachment. As stated by the authors, their coating method is applicable to other polymers, such as poly(lactic-co-glycolic acid) (PLGA).

By using a similar approach, in this work, a pressure-assisted method is developed to coat AM-manufactured PLA scaffolds with ceramic particles, intended for bone tissue engineering applications. Specifically, calcium carbonate (CaCO_3_) particles were used as the coating of the polymeric matrix. Our interest in the incorporation of CaCO_3_ is due to its capacity to act as a buffering agent to counteract the acidic degradation by products of PLA [41], since it buffers in the range of the physiological pH value (7.35–7.45) [42]. As for other ceramic additives, CaCO_3_ particles are expected to also have an effect on the degradation profile, surface, mechanical, and biological properties of the PLA matrix [25]. While HAp and β-TCP are the bioceramics that have attracted more attention in the BTE field (mainly due to their osteoconductivity and similar composition to that of bone) [43,44], several authors have previously evaluated the use of CaCO_3_ as an additive to improve the properties of PLA for bone tissue applications [26,45,46]. To the best of our knowledge, this is the first work in which CaCO_3_ particles are used to coat PLA scaffolds obtained by AM. In any case, the method developed can be applied to different bioceramics in combination with a polymeric matrix. In addition, it has been proven to be easy to implement as well as efficient, only requiring the use of a mould for the infiltration step, which is also relatively simple and fast. The coated scaffolds developed were evaluated in terms of the morphological, surface, degradation, and mechanical properties of the coated 3D structure.

## 2. Materials and Methods

### 2.1. Materials

Two commercial PLA filaments for 3D printing were used from Smart Materials 3D Printing S.L. (Alcalá la Real, Spain): Smartfil PLA (pure PLA filament) and Smartfil EP (PLA-based composite filament containing 30% *w/w* CaCO_3_). The latter was included in this study for comparison purposes, as the scaffolds of this group were manufactured from a commercial PLA filament that already includes in its formulation a relatively high content of CaCO_3_, instead of incorporating such ceramic particles as an outer coating. Therefore, only the pure PLA scaffolds manufactured were coated using the method proposed in this work. The CaCO_3_ used for ceramic coating (0179-500G, VWR) was acquired in powder form with a particle size below 30 μm.

### 2.2. Scaffold Manufacturing

The scaffolds developed were designed to be 9.8 mm in diameter and 7 mm in height by using computer-aided design software (Autodesk^®^ Fusion 360^®^, version 2.0.10806, Autodesk, Inc., San Rafael, CA, USA). The open-source Slic3r software (version 1.2.9, Free Software Foundation, Boston, MA, USA) was used to generate the G-code file of 3D structures with square-shaped pores, a rectangular 0/90° printing pattern, and a 50% theoretical porosity. The scaffolds were designed to have porosity and pore size within the optimal range for bone regeneration applications [25,47]. Scaffolds were manufactured by using a Hephestos 2 3D printer (BQ, Madrid, Spain), which is based on a MEX process, according to ISO/ASTM 52900:2021. Smartfil PLA and Smartfil EP filaments were fed to the 3D printer to produce PLA and PLA/CaCO_3_ composite scaffolds, respectively. Some of the main specifications of the Hephestos 2 3D printer, as well as the printing parameters used in the manufacture of PLA and composite scaffolds, are summarized in Table 1.

### 2.3. Application of the Ceramic Coating

**Figure 1:**
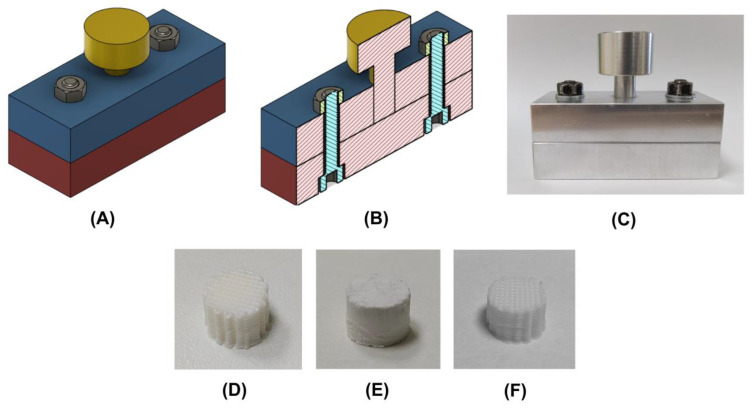
Three-part metallic mould manufactured for the application of the ceramic coating: (**A**) 3D model of the mould; (**B**) vertical section of the 3D model; (**C**) image of the assembled three-part mould; (**D**) PLA scaffold manufactured by material extrusion; (**E**) ceramic particles compacted around the scaffold; (**F**) coated scaffold obtained after the heating and washing steps.

A pressure-assisted method was developed to coat the PLA scaffolds with ceramic particles of CaCO_3_. A three-part mould was specifically designed for this purpose (Figure 1A,B) using Autodesk^®^ Fusion 360^®^ software. This device was manufactured with AW 5083 aluminium using milling and turning techniques, then assembled with steel bolts and nuts (Figure 1C). The cavity where the scaffolds were placed was 12 mm in diameter. The procedure for the application of the ceramic coating was as follows: after placing a PLA scaffold (Figure 1D) inside the mould, 1 mL of 60% *w/w* CaCO_3_ solution in distilled water was poured into the cavity, while trying to favour the infiltration of the ceramic slurry into the 3D structure. Then, the mould was closed by positioning the plunger, and the whole device was subjected to compression using a LI-1065 LIYI testing machine (Dongguan Liyi Environmental Technology Co., Ltd., Dongguan, China) in displacement control mode. The crosshead speed was set to 100 mm/min or 200 mm/min. The test was stopped when the applied force reached 1200 N, which translates to a final pressure of around 10 MPa. The criteria used to set these parameters are indicated in Section 4. This pressure allows the slurry to infiltrate the 3D structure, while being at the same time dehydrated. Consequently, the ceramic particles are compacted around the strands of the scaffold (Figure 1E). After the compression step, the coated scaffold was extracted from the mould and placed on a glass Petri dish, then transferred to an oven and kept at 155 °C for 45 min. By maintaining a temperature above the onset melting temperature of the base material (144.51 °C according to Figure S1 in Supplementary Materials), the polymeric surface enters a softened/molten state in which the CaCO_3_ particles can be embedded on the surface of the 3D structure. Additionally, it should be noted that the presence of the ceramic material around the scaffold allows us to preserve the designed structure of the scaffolds during the heat treatment step. Finally, the physically unattached ceramic particles were removed by washing the coated scaffolds with distilled water using an ultrasonic cleaner (30 min, 150 W, ULTR-2L0-001, Labbox). The coated scaffolds (Figure 1F) were left in a desiccator until completely drying before undergoing characterization.

As mentioned before, two different crosshead speeds were evaluated to investigate the effect of this parameter in the resulting ceramic coating. The coated groups of scaffolds tested are referred in the text to as 100S and 200S, which correlate with the crosshead speeds of 100 and 200 mm/min, respectively. On the other hand, non-coated PLA and PLA/CaCO_3_ composite scaffolds are referred as CN and COMPOSITE groups, respectively. As previously stated, COMPOSITE scaffolds are included in this work for comparison purposes; these scaffolds were manufactured using a PLA/CaCO_3_ filament, instead of incorporating said ceramic particles as an outer coating. In Table 2, a summary of the scaffold groups tested is presented.

### 2.4. Morphological Characterization of the 3D-Printed Scaffolds

Five replicas were tested per group of samples: CN, 100S, 200S, and COMPOSITE. Prior to the application of the ceramic coating, the scaffolds were morphologically characterized to ensure that there were non-significant differences between the different groups tested in terms of their main dimensions and weight. The weight of the structures was assessed by using a GR-200 analytical balance (±0.1 mg, A&D Instruments Ltd., Ahrensburg, Germany). Additionally, the height and diameter of the scaffolds, obtained as the mean value of 5 measurements per sample, were determined using an electronic calliper (±0.01 mm). After the coating process, the scaffolds were measured again following the same procedure to confirm that the pressure application and heating steps did not affect the overall dimensions of the 3D structure. The amount of ceramic additive incorporated into the scaffolds was calculated from the weight increase as a result of the coating process.

In addition, the pore size of the structures was determined, before and after coating, as the distance between strands (*n* = 9) by using an Olympus BX51 optical microscope (Olympus Co., Ltd., Tokyo, Japan). The thickness of the ceramic layer incorporated into the scaffolds surface was estimated as the difference between the results obtained from the pore size analysis before and after the coating process.

### 2.5. Surface Coating Evaluation

The effect of the ceramic coating on the hydrophilicity of the polymeric surface was evaluated by measuring the water contact angle (WCA) of coated and non-coated samples through the sessile drop method. Flat-surface samples obtained by compression moulding, as previously described [22,27], were used for this test. The samples had dimensions of 80 × 10 × 1 mm, and the manufacturing process was carried out in a Collin P200 P/M platen press (COLLIN Lab & Pilot Solutions GmbH, Maitenbeth, Germany). The ceramic coating was applied following the same procedure described for the scaffolds in Section 2.3. The WCA analysis was carried out at room temperature using an Ossila WCA measuring device (Ossila Ltd., Sheffield, UK). The static contact angle of 2 µL distilled water droplets (*n* = 10) was determined by using the open-source software ImageJ.

The surface of the coated and non-coated scaffolds was evaluated by scanning electron microscopy (SEM; Hitachi TM 3030 at an acceleration voltage of 15 kV). This equipment was coupled with an energy-dispersive X-ray (EDX) detector which allowed the assessment of the chemical composition of the samples in order to confirm the presence of CaCO_3_ on the ceramic-coated scaffolds. Prior to SEM analysis, the scaffolds were coated with Pd/Au for 2 min at 18 mA in an SC7620 sputter coater (Polaron, UK).

### 2.6. Mechanical Characterization

Coated and non-coated scaffolds were characterized under compression test using a LI-1065 LIYI testing machine (Dongguan Liyi Environmental Technology Co., Ltd., Dongguan, China) in displacement control mode. A compression load cell capacity of 500 kg was used, and a crosshead speed of 1 mm/min was set for the test. Four replicas of CN, 100S, 200S, and COMPOSITE scaffolds were tested. From the results obtained, the compressive modulus and offset compressive yield strength were calculated according to ISO 604. Thus, the compressive modulus was calculated from the initial steepest straight-line portion of the stress–strain curve, while the offset compressive yield strength was evaluated as the stress at which the stress–strain curve departs from linearity by 0.2% of deformation.

### 2.7. Degradation Study

An enzymatic degradation test was carried out to evaluate the effect of the CaCO_3_ coating on the degradation rate of the PLA-based scaffolds. Proteinase K enzymes from Tritirachium album (30 units per mg of protein, Merck, Darmstadt, Germany) were used at a concentration of 0.2 mg/mL in Trizma^®^ hydrochloride solution (pH 8.0, BioReagent, Merck). Sodium azide (ReagentPlus^®^, ≥99.5%, Merck, Darmstadt, Germany) was also added to the degradation media at the same concentration to diminish the risk of possible bacterial contamination. Four replicas of CN, 100S, 200S, and COMPOSITE scaffolds were tested. In addition, scaffolds immersed in degradation media without enzymes (one per group tested) were used as control samples. After measuring the weight of the scaffolds by using an analytical balance, a cleaning process under UV light for 30 min was applied. Then, the scaffolds were placed in a non-treated 24-well plate (Thermo Scientific™ Nunc™, Waltham, MA, USA) and 2 mL of degradation media were added per well. The well plate was maintained in an incubator at 37 °C for 5 days, and the degradation media was replaced daily. The pH of the media removed was measured (sensIONTM+PH1, ±0.01, HACH) to obtain the pH profile from each group of scaffolds during the test. After 5 days, the scaffolds were washed with distilled water and allowed to dry until they reached constant weight to determine the weight loss of the degraded 3D structures.

### 2.8. Statistical Analysis

The statistical analysis was carried out using MATLAB software (MATLAB and Statistics Toolbox Release 2021a, The MathWorks, Inc., Natick, MA, USA). The Wilcoxon two-sided rank sum test was used when two groups were compared, while the Kruskal–Wallis test and a subsequent multiple comparison test were used when data from more than two groups were analysed. The significance level was set to * *p* < 0.05 and ** *p* < 0.01, for statistically significant and highly statistically significant differences, respectively. All figures and tables show the mean values obtained for each group tested. Standard deviations are represented with error bars in the case of figures.

## 3. Results

### 3.1. Morphological Characterization

The comparison of the PLA groups of scaffolds in terms of their height, diameter, and weight revealed non-significant differences between them before the coating process. For these groups, the coefficients of variation were below 0.9%, 1.0%, and 0.4% for their weight, height, and diameter, respectively. On the other hand, as expected, a highly significant difference (*p* < 0.01) was found when comparing the manufactured PLA scaffolds with the COMPOSITE ones, as the latter included in their formulation a 30% *w/w* ceramic additive. The coefficients of variation of the COMPOSITE scaffolds were 1.0%, 0.6%, and 0.2% for the weight, height, and diameter evaluations, respectively.

The application of the ceramic coating procedure detailed above did not show a significant effect (*p* > 0.05) on the overall dimensions (height and diameter) of the 3D structures, nor were there any statistical difference between the final morphological characteristics of the 100S and 200S coated groups. In contrast, the weight of the scaffolds increased significantly (*p* < 0.01) after the incorporation of CaCO_3_ particles into the PLA matrix. For the 100S group, the amount of ceramic additive embedded represented around 7.7% of the final weight of the scaffolds. On the other hand, this value was around 6.0% for the 200S scaffolds. Notably, the incorporation of CaCO_3_ particles increased the mean weight in both cases to such an extent that the highly significant (*p* < 0.01) difference that was found before coating when compared to the COMPOSITE control group was no longer observed. However, a highly statistically significant difference (*p* < 0.01) was found when comparing the weight of the 100S group to the CN group of scaffolds.

**Figure 2:**
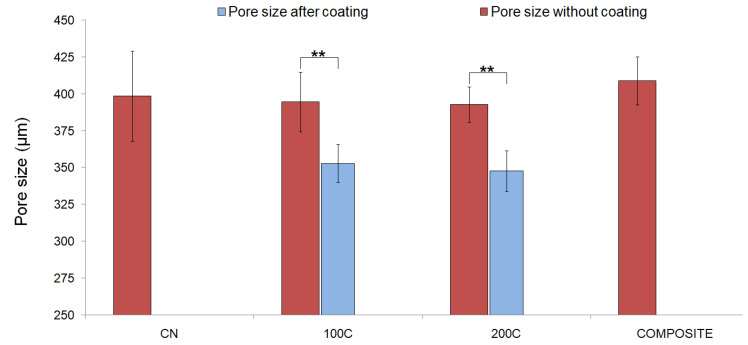
Pore size values of the 3D-printed scaffolds with and without CaCO_3_ coating (** *p* < 0.01).

Regarding the pore size assessment that was carried out (Figure 2), no statistical differences were obtained when comparing the four groups tested before applying the coating procedure. The average value of the pore size of the manufactured scaffolds, estimated as the distance between filaments, was 399 ± 20 µm. This value is in the optimal range for bone regeneration applications (300–500 µm [47]). After the ceramic coating, the 100S and 200S groups showed a significant decrease (** *p* <0.01) in pore size in comparison to their initial values, which correlates with the incorporation of CaCO_3_ particles to the polymeric surface in the form of a thin layer. Mean pore sizes of 353 ± 13 µm and 348 ± 14 µm were obtained for the 100S and 200S groups of scaffolds, respectively. As a result, the estimated thickness of the ceramic coating was determined to be in the range of 10–30 µm for the coated groups of scaffolds, which is in accordance with the maximum particle size (30 µm) of the CaCO_3_ particles used.

### 3.2. Surface Properties

**Figure 3:**
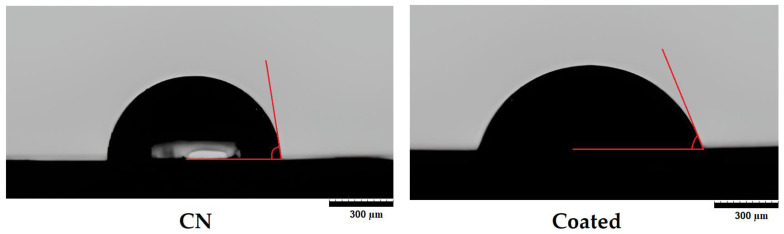
Water contact angle of non-coated (CN) and coated samples determined by the sessile drop method.

**Figure 4:**
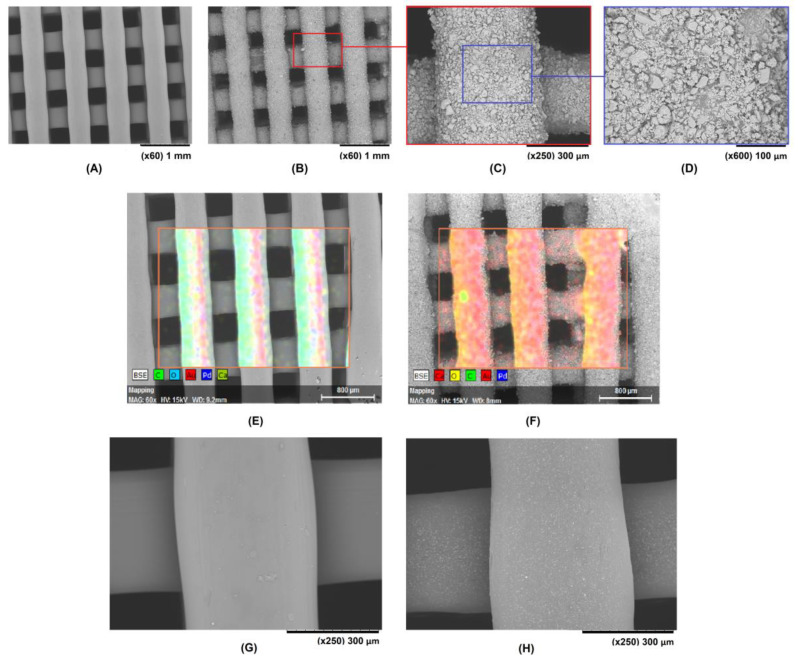
SEM images of CN (**A**) and 100S (**B**–**D**) scaffolds at various magnifications. EDX mapping of CN (**E**) and 100S (**F**) scaffolds. A comparison between the surfaces of CN (**G**) and COMPOSITE (**H**) scaffolds is also included.

The WCA of the base material was significantly reduced by applying the proposed coating method (Figure 3): the initial value of 87.7 ± 1.4° for the non-coated samples decreased to a mean value of 82.6 ± 4.2° for the 100S scaffolds (*p* < 0.05) and 82.9 ± 5.3° for the 200S scaffolds (*p* < 0.05). The decrease in the WCA in the coated samples could be related to the increased surface roughness of the scaffolds, as confirmed by the SEM analysis (Figure 4). The ceramic coating layer incorporated into the surface of a 100S scaffold is clearly shown in Figure 4B,C, with the CaCO_3_ particles evenly distributed throughout the whole surface of the 3D structure.

The presence of the ceramic material was also confirmed by EDX when comparing the surface of a CN scaffold (Figure 4E) with the surface of a 100S scaffold (Figure 4F). No Ca was detected in the former, and it is highlighted in red for the latter. In addition, the surface morphology of a COMPOSITE scaffold is shown in Figure 4H. A good distribution of CaCO_3_ is observed, with the EDX detector also confirming the presence of Ca.

### 3.3. Mechanical Properties

**Figure 5:**
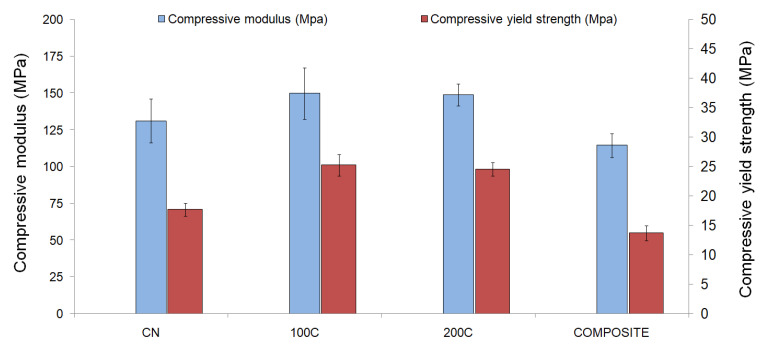
Mechanical properties of the non-coated and ceramic-coated scaffolds under compression testing.

The results obtained from the mechanical characterization of the samples are presented in Figure 5 and Table 3. Additionally, as an example, the stress–strain diagram of one of the 100S scaffolds developed is shown in Figure S2 in the Supplementary Materials, where the region used for calculating the compression modulus is highlighted.The mean values of the compressive modulus were as follows: 131 ± 7 MPa for the CN scaffolds, 150 ± 2 MPa for the 100S scaffolds, 149 ± 4 MPa for the 200S scaffolds, and 115 ± 2 MPa for the COMPOSITE scaffolds. These values are in the reported range for cancellous bone [48,49]. The incorporation of CaCO_3_ particles significantly increased the compressive modulus, with the 100S and 200S groups showing statistically significant differences (*p* < 0.05) in comparison to the CN group. In contrast, the COMPOSITE scaffolds showed the lowest values among the groups tested, as the relatively high content of ceramic material led to a significant decrease (*p* < 0.05) in the compressive modulus. When comparing the coated scaffolds with the COMPOSITE group, statistically significant differences (*p* < 0.05) were also obtained.

Similar conclusions were drawn from the compressive yield strength assessment (Figure 5 and Table 3), as once again the coated scaffolds were superior to the CN group (*p* < 0.05 for both the 100S and 200S groups). In this case, the results were as follows: 18 ± 2 MPa for the CN scaffolds, 25 ± 1 MPa for the 100S scaffolds, 25 ± 2 MPa for the 200S scaffolds, and 14 ± 1 MPa for the COMPOSITE scaffolds. As also observed for the compressive modulus, non-significant differences were obtained between the 100S and 200S scaffolds in terms of the compressive yield strength, while the COMPOSITE group was significantly inferior (*p* < 0.05) compared to any other group tested.

### 3.4. Degradation Test Results

**Figure 6:**
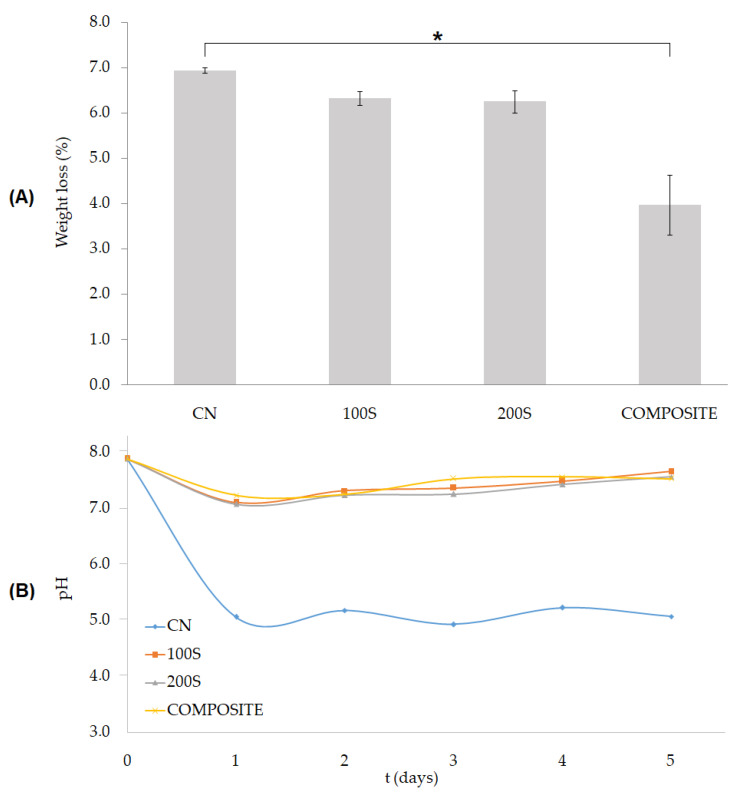
Results from the enzymatic degradation test: (**A**) weight loss (%) of the scaffolds after five days (* *p* < 0.05); (**B**) pH variation in the degradation media of each group tested.

The weight loss of each group of scaffolds after five days of enzymatic degradation is shown in Figure 6A. Non-significant differences (*p* > 0.05) were obtained when comparing the CN, 100S, and 200S groups, which showed weight reductions of 6.9 ± 0.1%, 6.3 ± 0.1%, and 6.3 ± 0.2%, respectively. The slightly lower degradation rate of the ceramic-coated scaffolds (the 100S and 200S groups) could be related to the lower polymeric surface available for enzymatic attack compared to that of non-coated PLA scaffolds (the CN group). On the other hand, a value of 4.0 ± 0.7% was obtained in the case of the COMPOSITE group, which resulted in a statistically significant difference (* *p* < 0.05) compared to the CN group. The weight of the scaffolds used as the control (one per group, which were immersed in a buffer solution without enzymes) showed no significant variation after five days.

Despite showing a similar mean weight loss to the one obtained for the CN group, the 100S and 200S coated scaffolds were able to maintain the pH of their surrounding media at the same level to that of the COMPOSITE group. After five days, the pH values obtained for each group were as follows: 5.07 ± 0.1 for the CN, 7.66 ± 0.1 for the 100S, 7.57 ± 0.1 for the 200S, and 7.53 ± 0.1 for the COMPOSITE scaffolds. According to the results, the release of CaCO_3_ particles into the media proved to be an effective method to counteract the pH decrease in the media caused by the presence of PLA acidic byproducts. Although the ceramic particles were found in a lower concentration in the 100S and 200S scaffolds (6.0–7.7% *w/w*) compared to that of the COMPOSITE group (30% *w/w*), the fact that they were located on the surface of the 3D structure led to a rapid release during the first degradation steps. In this way, the pH of the surrounding media could be maintained within the physiological range [42]. In contrast, the pH of the media of the CN group already decreased to a value of 5.06 ± 0.1 after one day of enzymatic degradation, then remained at that level until the end of the test. The abrupt decrease in the pH could have generated a strong inflammatory response in the surrounding tissues and hindered tissue regeneration [9].

## 4. Discussion

The coating method presented in this work has been proven to be effective in coating 3D-printed PLA scaffolds. Additionally, it has the potential to be applied to other complex structures, polymeric materials, and ceramic additives. Apart from being relatively fast and easy to implement, this method does not require the use of toxic organic solvents, whose potential residue can hinder medical applications [50]. Noteworthy, the pressure-assisted method developed also offered great repeatability, as demonstrated by the low variation factor (the sampling standard deviation divided by the mean value) obtained when calculating the final pore size, the percentage of coating, or the compressive modulus of the coated scaffolds, which, in all of these cases, was lower than 5%.

**Figure 7:**
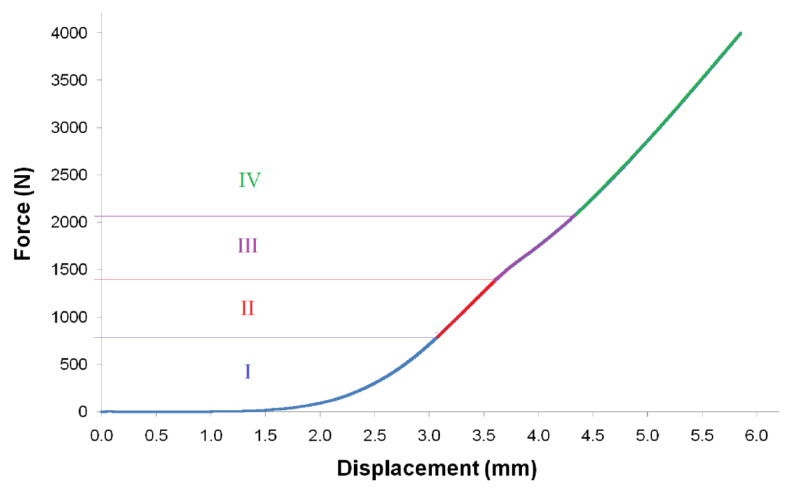
Force-displacement curve obtained by running the coating method using a compressive force up to 4000 N and identification of different process steps.

Regarding the process parameters, enough force must be applied at a relatively high speed to ensure not only that the ceramic slurry infiltrates the pores of the 3D-printed structure, but that it is also dehydrated. In this way, the ceramic particles are compacted around the polymeric strands. As illustrated in Figure 7, the force-displacement graph of a PLA scaffold subjected to the coating method under compression shows four different regions: (I) the toe region, which does not represent any property of the material; (II) the linear region, where the compression modulus is calculated; (III) the region above compressive yield point, in which complete dehydration takes place [34]; and (IV) the second linear region, where the further compaction of the CaCO_3_ particles occurs. The PLA scaffolds tested using a maximum force greater than the compressive yield point showed significant deformations of their structure. For scaffolds obtained by AM techniques for a custom-tailored design, maintaining the overall characteristics of the produced 3D structure is particularly important. Thus, a force of 1200 N (in region II of Figure 7) was set as the maximum target value for the coating process of the 3D-printed PLA scaffolds used in this work (see Figure S2 in Supplementary Materials for more reference). Regardless of their design or the base material used, this decision process to select the appropriate force (or pressure) can be applied to other scaffolds by simply determining their main mechanical characteristics first.

The selection of the compression speed also plays an important role in the coating process, as higher values allow for a better infiltration of the ceramic slurry into the 3D structure. In addition, while the force applied (1200 N) did not generate the complete dehydration of the slurry, it led to a partial water loss and a certain degree of ceramic particle compaction. We hypothesized that the higher the speed, the better the particles would be compacted and pressed against the polymeric surface, thus improving the effectiveness of the embedding process subsequently applied by heating. According to the results obtained in the preliminary tests carried out, low crosshead speeds (such as 1 and 5 mm/min) led to the poor infiltration of the slurry and scarce CaCO_3_ coatings, as confirmed by the weight change in the scaffolds and the analysis of their surface by optical microscopy. Although the maximum force reached during infiltration was maintained, low compression speeds may lead to higher dehydration as the test takes more time, thus favouring water removal. Consequently, in the final part of the test, the slurry is more viscous for low speeds, thus hindering the infiltration. By increasing the compression speed, the amount of ceramic particles incorporated into the polymeric scaffold increased, but only until a speed of 100 mm/min. From this value, the infiltration and compaction of the particles was not significantly improved, as demonstrated by the results obtained in this work: non-statistically significant differences were obtained between the 100S and 200S groups in terms of coating thickness, surface hydrophilicity, or their mechanical properties. Similar results were also obtained for scaffolds tested at 400 mm/min (the results of these preliminary tests are not shown in this paper). In contrast to an increase in maximum force, the use of a higher compression speed did not result in the deformation of the 3D structures, as was expected.

As stated in Section 2.3., after infiltrating the ceramic slurry by compression, the scaffold was extracted from the mould and transferred to an oven for the heating step of the coating method. If the compaction of the CaCO_3_ particles around the polymeric strands was successful, a 3D ceramic cover formed (corresponding to the negative of the scaffold geometry with the external dimensions of the mould, as shown in Figure 1E), which helps support the scaffold during heating [34], thus keeping dimensional changes to a minimum. The temperature of the oven was set after a differential scanning calorimetry (DSC) analysis of a Smartfil PLA sample (see Figure S1 in Supplementary Materials). An onset temperature of 144.51 °C was obtained, while the peak melting temperature was determined to be 150.43 °C. Thus, the temperature of the oven had to be set to over 145 °C to ensure PLA melting. As the ceramic cover hinders the heat flow to the scaffold, temperatures above 150 °C were necessary to effectively melt the polymeric matrix. However, excessive temperatures led to heat-induced deformations. An optimal temperature of 155 °C was established to allow PLA melting while maintaining the scaffolds’ overall dimensions.

Apart from temperature, time also had a major effect on the amount of ceramic particles embedded into the matrix. Giving more time for the molten polymer to diffuse in between the compacted particles allows a greater number of particles to be physically attached to the PLA surface. A time of 45 min was chosen in this study; under these conditions, the generated ceramic layer reduced the initial pore size of the scaffolds (399 ± 20 µm) to values still in the optimal range reported for cancellous bone (300–500 µm [47]): 353 ± 13 µm and 348 ± 14 µm for the 100S and 200S scaffolds, respectively. The coated PLA scaffolds subjected to 1 h of heating treatment, however, showed a mean pore size lower than 300 µm, which could hinder the application of these scaffolds for bone regeneration due to limited vascularization [51]. In any case, since temperature and time are parameters that can be easily tuned, the proposed method can be applied to any type of polymeric scaffold to be coated (even with a wide range of additives) as well as allows one to control, up to a certain extent, the amount of coating. Therefore, the robustness and flexibility of this coating method could be useful for multiple applications.

**Figure 8:**
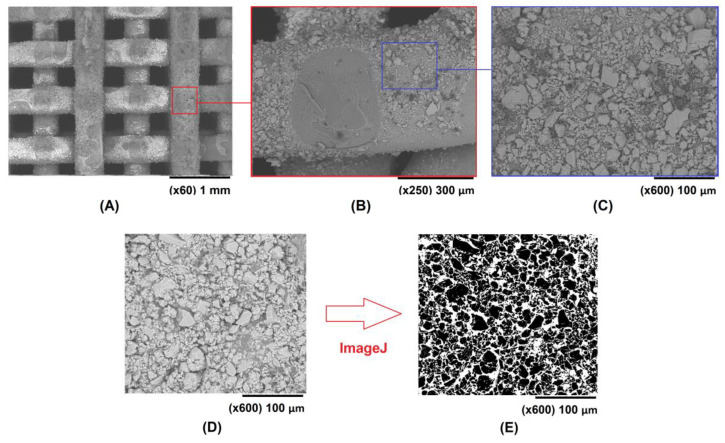
SEM images of the interior of a 100S scaffold (**A**–**C**) at various magnifications. Example of image treatment using ImageJ software to estimate the percentage of surface area coated (**D**,**E**).

While SEM images of the top layer of the scaffolds are shown in Figure 4, the inner part of them is presented in Figure 8A–C. The samples were cut transversely to address the ceramic coating inside the 3D structure, then sputter-coated with Pd/Au for the SEM observation. These images confirmed that the ceramic particles are present in the entire available surface of the scaffold. Uncoated areas of the PLA surface, such as the one shown in Figure 8B, are due to the prior existence, in that particular area, of a strand from the immediately upper layer (before performing the cut on the 3D structure). To estimate the percentage of PLA scaffold surface covered by the CaCO_3_ particles, the public domain ImageJ software [52] was used to analyze the SEM images obtained for the 100S and 200S scaffolds at ×600 magnification. The image analysis was performed after adjusting the threshold for the adequate identification of the polymer and ceramic particles present in the images (Figure 8D,E). The results showed that the PLA surface was about 61% coated. For these CaCO_3_ coatings, a strong bonding interface was achieved, as 30 min of sonication using an ultrasonic cleaner was insufficient to remove the ceramic particles firmly attached to the polymeric matrix. The stability of the coating in an aqueous medium was also evaluated by analysing the control samples of the enzymatic degradation study, since they were the coated and uncoated scaffolds that were submerged in a buffer solution for five days. The final weight of the scaffolds showed that there was no significant loss of ceramic material.

As mentioned above, the amount of ceramic particles embedded in the polymeric surface can be increased by adjusting the process parameters (mainly the temperature and time of the heating step), but only up to a limit imposed by the available surface area of the scaffold. The coating thickness is also constrained by the need to maintain the pore size of the structure within a certain range to allow for vascularization and tissue ingrowth [51,53]. On the contrary, a higher ceramic content could be achieved by using the particles as a filler or additive to the PLA matrix, finding in this case the restriction in the processability of the composites by the specific manufacturing technique to be used. Furthermore, a relatively high content of ceramic additives could affect the mechanical properties of the scaffold [16,27,54]. Esposito Corcione et al. [16], for example, obtained a reduction in the Young’s modulus by almost half when testing 3D-printed PLA scaffolds (238.98 ± 19.05 MPa) and 50% *w/w* PLA/HAp composite scaffolds (124.04 ± 25.21 MPa). The authors attributed the observed loss of mechanical properties to the increased micro- and macroporosity of the composite scaffolds due to the high content of HAp. In this work, we characterized, under compression, PLA (CN group) and PLA-based scaffolds with 30% *w/w* CaCO_3_ particles (the COMPOSITE group), and the results also showed a significant decrease (*p* < 0.05) in the compressive modulus due to the presence of the ceramic additive: 131 ± 7 MPa for the CN scaffolds and 115 ± 2 MPa for the COMPOSITE scaffolds. On the other hand, the application of the coating method led to an increase in the mechanical properties of around 14% for the compressive modulus and 41% for the compressive yield strength, compared to those of the uncoated PLA scaffolds. This increase in the compressive modulus and yield strength was expected as the coating process is adding new material to the 3D-printed samples. Therefore, for approximately the same external dimensions, the equivalent mechanical properties were improved.

The modifications introduced on the polymeric surface due to the ceramic coating also affect the degradation profile of the scaffolds. The adsorption of the enzymes on the PLA surface, which is the first and one of the key steps of the degradation process, can be altered by the presence of the CaCO_3_ particles as well as the enhanced surface roughness and hydrophilicity that they generate, thus potentially affecting the enzymatic degradation mechanism of PLA [55]. In this way, the degradation rate of the 100S and 200S scaffolds could have been partially improved during the first steps of degradation as a consequence of their higher surface roughness (as illustrated in Figure 7) and improved hydrophilicity (Figure 3).

When the enzymatic degradation progresses, it follows a surface erosion mechanism [56]: as the surface roughness is enhanced and new regions of the polymer are exposed to the enzymes due to the chains’ cleavage reactions, the degradation rate of the PLA-based scaffolds is accelerated. The increased break of ester bonds leads to a decrease in the weight of the scaffolds, the release of low-molecular-weight fragments and acidic byproducts, and a subsequent decrease in the pH of the surrounding media [57]. This is the case for the CN group of scaffolds, for which the highest degradation rate (6.9 ± 0.1%) and the lowest pH of the media (5.07 ± 0.1) after five days of enzymatic degradation were obtained. In contrast, the COMPOSITE group showed a pH in the range of 7.24–7.53 during the test, with a mean weight reduction of only 4.0 ± 0.7% after five days (compared to the CN scaffolds, * *p* < 0.05). From these results, it can be concluded that the ceramic particles released were able to counteract the pH decrease due to the PLA degradation byproducts. However, at the same time, the maintenance of the pH level hindered the autocatalytic degradation of the polymer, then reducing the degradation rate of the scaffolds.

Noteworthy, PLA scaffolds coated with CaCO_3_ particles (the 100S and 200S groups) also showed the capacity to maintain the pH level of the media within the physiological range [42] during the test, while being degraded at a rate similar to that of the CN group (*p* > 0.05). Ceramic particles are released during the first steps of degradation to counteract the acidic byproducts of PLA, thus exposing a polymeric surface with an enhanced roughness that degrades at a rapid rate in comparison to that of the COMPOSITE group. The fact that the ceramic particles can be incorporated into the PLA matrix without reducing the degradation rate but altering the pH profile shows the potential of the developed coated scaffolds for BTE applications. If a higher degradation rate of the scaffolds is needed to match new bone tissue ingrowth, additional strategies for the incorporation of the ceramic coating can be applied, including the following: the selection of a polymer with a low molecular weight [12], the adjustment of L- and D-enantiomer content [58],the modification of the initial degree of crystallinity [59], and other surface modifications (e.g., plasma or alkali treatments) [19].

In addition to the biocompatibility enhancement generated by the incorporation of ceramic materials as a coating of the polymeric matrix [60,61,62], the observed increase in the surface roughness (Figure 4A,B), coupled with the decrease in its hydrophobicity (Figure 3), could lead to the improved metabolic activity of the bone cells adhered to the coated scaffolds [27,63,64]. Furthermore, cell growth is not hindered by the reduction in the pH of the media surrounding the PLA-based scaffold, due to the buffer effect of the ceramic particles released during the degradation process of the 3D structure. While the biological evaluation of the developed coated scaffolds is out of the scope of this work, numerous previously published works have already revealed that ceramic coatings effectively enhance the cell adhesion, proliferation, and differentiation of bone cells [65,66,67,68]. Although the osteoinduction mechanism of ceramic compounds is still unclear [69], surface topography, surface chemistry, and crystallinity are among the parameters that have been demonstrated to have an effect on cell response [70,71,72]. The expected biological properties’ enhancement, together with the increase in mechanical properties and the concentration of the functionality improvement at the cell–surface interface, make the coating method developed in this work a strategy with great potential to be further evaluated for bone regeneration applications.

## 5. Conclusions

The pressure-assisted and heat-induced coating method presented in this work (the infiltration of a 60% *w/w* CaCO_3_ solution in 3D-printed PLA scaffolds by compression at 100–200 mm/min up to 1200 N load, followed by a subsequent heat treatment at 155 °C for 45 min and final cooling and washing step) has proven to be effective for embedding CaCO_3_ particles on the surface of PLA scaffolds intended for BTE applications. The ceramic coating process did not affect the overall dimensions of the AM-manufactured scaffolds. A statistically significant weight increase (6.0–7.7%) was obtained due to the incorporation of the ceramic particles, which were evenly distributed throughout the polymeric surface to cover around 61% of it. The thin layer of ceramic coating generated on the PLA scaffolds (10–30 µm) provided a significant increase in the mechanical properties, with a compressive modulus of around 150 MPa, which is in the range of values reported for cancellous bone. The coated scaffolds showed a degradation rate under enzymatic conditions similar to that of pure PLA scaffolds, but their pH profile confirmed the capacity of the released ceramic particles to counteract the pH decrease caused by the polymer degradation (the pH of the surrounding media was maintained within the physiological range). Furthermore, the increased surface roughness and improved hydrophilicity of the coated scaffolds (statistically significant reduction in the contact angle from 87.7 ± 1.4° for the non-coated samples to a mean value of 82.6–82.9° for the coated ones) have a positive effect on the cell adhesion and proliferation of bone-like cells.

The developed ceramic coating method was applied using 3D-printed PLA scaffolds and CaCO_3_ coating, which has not been reported yet in the literature. However, the coating method is applicable to different polymeric materials and scaffold designs, even with different coating materials, with a very simple and relatively fast procedure. Apart from this, the process parameters (mainly temperature and time) can be adjusted to control, up to a certain extent, the amount of coating added to the surface, thus allowing for the production of coated scaffolds with suitable properties for BTE applications.

## Figures and Tables

**Table 1:** Specifications of the BQ Hephestos 2 3D printer and printing parameters.

	Description
**Specifications**	
printing speed	up to 200 mm/s
layer resolution	50 µm
printable area	210(x) × 297(y) × 220(z) mm
heater cartridge	40 W, 12 V
liquefier temperature	up to 250 °C
cooling	axial fan and cooling blower
**Printing parameters**	
printing pattern	rectangular 0/90°
nozzle diameter	0.40 mm
layer height	0.30 mm
extrusion width	0.48 mm
extrusion speed	40 mm/s
liquefier temperature	215 °C

**Table 2:** Description of groups of scaffolds tested and number of replicas.

Group	Base Material	Coating	Description	Replicas
CN	Smartfil PLA	-	Pure PLA scaffolds	10
100S	Smartfil PLA	CaCO_3_	Crosshead speed set at 100 mm/min for infiltration	10
200S	Smartfil PLA	CaCO_3_	Crosshead speed set at 200 mm/min for infiltration	10
COMPOSITE	Smartfil EP	-	Composite PLA/CaCO_3_ (70/30% *w/w*) scaffolds	10

**Table 3:** Significance level of the differences found in the statistical analysis of mechanical test results (* *p* < 0.05). Results related to the compressive modulus are highlighted in blue, while the ones related to compressive yield strength are marked in red.

Group	CN	100S	200S	COMPOSITE
CN		*	*	*
100S	*		**-**	*
200S	*	**-**		*
COMPOSITE	*	*	*	

## Data Availability

The data that support the findings of this study are available from the corresponding author upon reasonable request.

## Supplementary Materials

The following supporting information can be downloaded at: https://www.mdpi.com/article/10.3390/polym15112506/s1, Figure S1: DSC results obtained when analyzing a sample of Smartfil PLA; Figure S2: Stress–strain diagram obtained from the mechanical test of a 100S scaffold under compression.
