# Supplementary material for: Calcium Carbonate Coating of 3D-Printed PLA Scaffolds Intended for Biomedical Applications
Source: Polymers (Basel). 2023 May 29;15(11):2506. doi: 10.3390/polym15112506 (PMC10255753; doi:10.3390/polym15112506)
Supplement: Supplementary file 1 [file polymers-15-02506-s001.zip › polymers-2346131-supplementary.pdf]

Differential Scanning Calorimetry (DSC) analysis

A sample of Smartil PLA was subjected to DSC analysis in a differential scanning calorimeter DSC 4000 (Perkin Elmer, Waltham, MA, USA). A small fragment (~10 mg) of the Smartfil PLA filament was cut and placed in an aluminum crucible, then sealed and subjected to a heating/cooling/heating cycle from 30–230°C. Other parameters include a nitrogen flow of 20 mL/min and heating and cooling rates of 10°C/min. The onset temperature (at which melting process starts), the peak melting temperature and the melting enthalpy (Figure S1) were determined from the data of the second heating step.

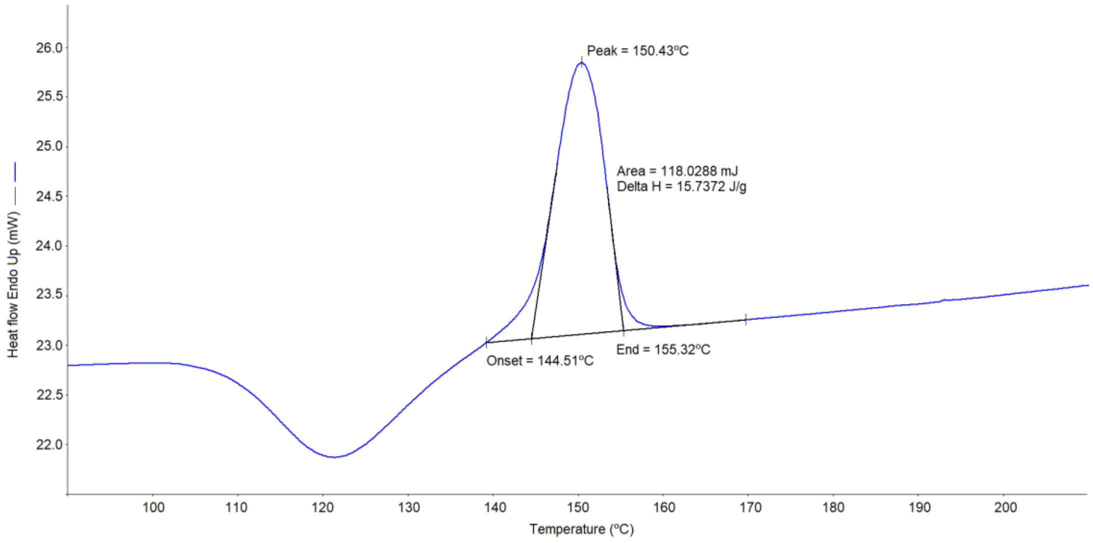

Figure S1. DSC results obtained when analyzing a sample of Smartfil PLA.

Mechanical characterization of the developed scaffolds: stress-strain diagram

Figure S2 shows the stress-strain diagram of a 100S scaffold tested under compression using a LIYI testing machine (LI-1065, Dongguan Liyi Environmental Technology Co., Ltd., China) in displacement control mode. A compression load cell capacity of 500 kg was used, and a crosshead speed of 1 mm/min was set for the test. From the results obtained, the compressive modulus and offset compressive yield strength were calculated according to ISO 604. In Figure S2, the result obtained for the compressive modulus is shown, as well as the region used to determine such mechanical property.

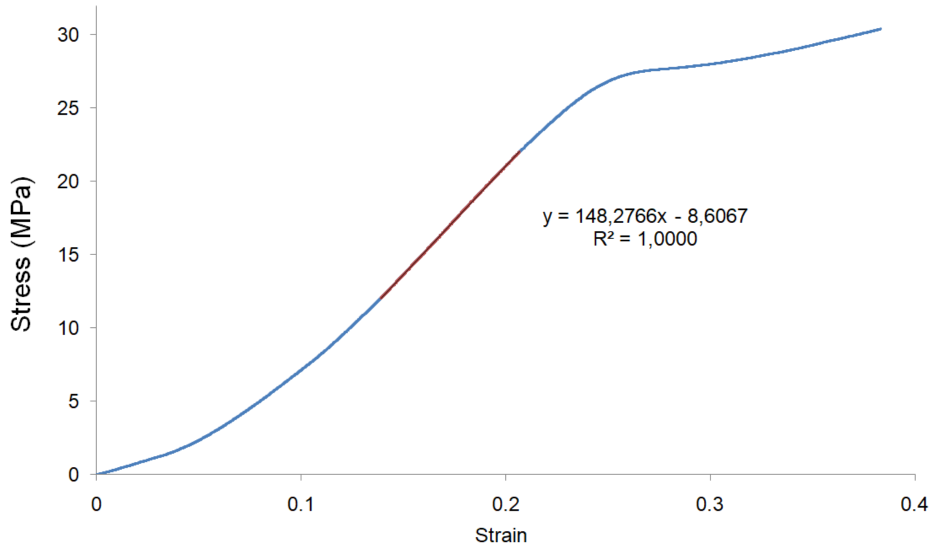

Figure S2. Stress-strain diagram obtained from the mechanical test of a 100S scaffold under compression.
